# Effects of a New Form of Resistance-Type High-Intensity Interval Training on Cardiac Structure, Hemodynamics, and Physiological and Performance Adaptations in Well-Trained Kayak Sprint Athletes

**DOI:** 10.3389/fphys.2022.850768

**Published:** 2022-03-10

**Authors:** Mohsen Sheykhlouvand, Hamid Arazi, Todd A. Astorino, Katsuhiko Suzuki

**Affiliations:** ^1^Department of Exercise Physiology, Faculty of Sport Sciences, University of Guilan, Rasht, Iran; ^2^Department of Kinesiology, California State University, San Marcos, CA, United States; ^3^Faculty of Sport Sciences, Waseda University, Tokorozawa, Japan

**Keywords:** oxygen consumption, cardiorespiratory fitness, intermittent exercise, water sports, exercise training

## Abstract

This study examined the effects of a resistance-type high-intensity interval training (RHIIT) matched with the lowest velocity that elicited $\dot{\text{V}}$O_2peak_ (100% *v*$\dot{\text{V}}$O_2peak_) in well-trained kayak sprint athletes. Responses in cardiac structure and function, cardiorespiratory fitness, anaerobic power, exercise performance, muscular strength, and hormonal adaptations were examined. Male kayakers (*n* = 24, age: 27 ± 4 years) were randomly assigned to one of three 8-wk conditions (*N* = 8): (RHIIT) resistance training using one-armed cable row at 100% *v*$\dot{\text{V}}$O_2peak_; paddling-based HIIT (PHIIT) six sets of paddling at 100% *v*$\dot{\text{V}}$O_2peak_; or controls (CON) who performed six sessions including 1-h on-water paddling/sessions at 70–80% maximum HR per week. Significant increases (*p* < 0.05) in $\dot{\text{V}}$O_2peak_, *v*$\dot{\text{V}}$O_2peak_, maximal cardiac output, resting stroke volume, left ventricular end-systolic dimension, 500-m paddling performance were seen pre- to post-training in all groups. Change in $\dot{\text{V}}$O_2peak_ in response to PHIIT was significantly greater (*p* = 0.03) compared to CON. Also, 500-m paddling performance changes in response to PHIIT and RHIIT were greater (*p* = 0.02, 0.05, respectively) than that of CON. Compared with pre-training, PHIIT and RHIIT resulted in significant increases in peak and average power output, maximal stroke volume, end-diastolic volume, ejection fraction, total testosterone, testosterone/cortisol ratio, and 1,000-m paddling performance. Also, the change in 1,000-m paddling performance in response to PHIIT was significantly greater (*p* = 0.02) compared to that of CON. Moreover, maximum strength was significantly enhanced in response to RHIIT pre- to post-training (*p* < 0.05). Overall, RHIIT and PHIIT similarly improve cardiac structure and hemodynamics, physiological adaptations, and performance of well-trained kayak sprint athletes. Also, RHIIT enhances cardiorespiratory fitness and muscular strength simultaneously.

## Introduction

High-intensity interval training (HIIT), repeated bouts at near-maximal to maximal intensities interspersed with recovery, has been shown to increase maximum oxygen uptake ($\dot{\text{V}}$O_2max_), anaerobic power, and exercise performance (Laursen and Buchheit, 2019; Mallol et al., 2020). Optimizing exercise programs leading to these adaptations can be complex and involve modification of intensity, frequency, and duration, during prescription (MacInnis and Gibala, 2017). In addition, the modality of the exercise plays a key role in designing specialized programs (Fereshtian et al., 2017; Sheykhlouvand et al., 2018a).

Kayak sprint is an Olympic event and takes place on a flat-water course and races are contested by kayak. In a kayak, the athlete competes in a sitting position using a double-blade paddle. At the international level, the discipline is competed at four distances from 200 to 5,000-m, both individually and in crew of up to four. Kayak individual events include the 200-m (∼38 s), 500-m (∼100 s) and 1,000-m (∼220 s) for world-level kayakers (International Canoe Federation).^1^ To compete at this level, kayak sprint athletes need substantial upper-body aerobic and anaerobic power (Bishop, 2000; Michael et al., 2008; Zouhal et al., 2012; Borges et al., 2015; Sheykhlouvand et al., 2015; Sheykhlouvand and Forbes, 2017; Barzegar et al., 2021). For example, contribution of aerobic metabolism during kayak sprinting in elite athletes has been estimated using the accumulated oxygen deficit method to be ∼37, 64–78, and 85–87% for 200, 500, and 1,000-m events, respectively. Contrary to 500 and 1,000-m performance, 200-m race performance is not related to maximum oxygen uptake ($\dot{\text{V}}$O_2max_) but lactate threshold and anaerobic capacity/power (Paquette et al., 2018). On the other hand, upper-body strength and muscular endurance are strong determinants of kayak sprint performance, and augmenting the pulling motion enhances the pulling force throughout the pull phase of paddling and improves maintenance of speed (Ualí et al., 2012; McKean and Burkett, 2014). As the concurrent action of several physiological variables affects sport-specific performance (Sousa et al., 2020), kayak sprint athletes need to emphasize these factors in their programs to maximize exercise performance. Although paddling technique and economy play an important role in athletic performance, in this study we specifically focused on physical and physiological variables.

Kayak sprint paddlers often need to reach peak performance for competitions several times over an annual training cycle and require a training program to achieve fitness in a short period of time. Training programs capable of improving both metabolic conditioning and muscular strength are time-demanding (Haff and Triplett, 2016; Sheykhlouvand et al., 2016b) and cannot be prioritized over each other in kayak sprint. It is therefore common practice for athletes to engage in resistance training in combination with training aimed at enhancing cardiorespiratory and metabolic fitness (e.g., HIIT). In such situations, designing a sport-specific time-efficient training protocol with a combination of resistance and aerobic training capable of satisfying both ends of the strength-endurance continuum could be of value.

*v*$\dot{\text{V}}$O_2peak_ is known as an optimal load to stimulate cardiorespiratory fitness adaptations (Buchheit and Laursen, 2013) and we recently showed that paddling-based HIIT using a kayak ergometer at the lowest velocity that elicited $\dot{\text{V}}$O_2peak_ (100% *v*$\dot{\text{V}}$O_2peak_) improves cardiorespiratory fitness and anaerobic power in trained paddlers (Sheykhlouvand et al., 2016a,b, 2018b). Ualí et al. (2012) demonstrated that one-armed cable row exercise may stress the muscles involved in kayak paddling, so in this study, we employed this modality to implement a new resistance-type HIIT regimen (RHIIT) matched with 100% *v*$\dot{\text{V}}$O_2peak_ to perform a single-mode training to simultaneously stimulate upper-body muscular strength and cardiorespiratory fitness adaptations. In other words, we may perform a single-mode exercise to stimulate the adaptations related to both qualities instead of performing two isolated sessions for each training mode. Hence, we decided to test: (a) whether RHIIT might be considered as an effective stimulant as traditional HIIT to improve cardiorespiratory fitness and (b) if RHIIT may enhance both cardiorespiratory fitness and muscular strength simultaneously. Accordingly, the aim of this study was to investigate the effects of an 8-week resistance-type HIIT on cardiac structure and hemodynamics, aerobic and anaerobic power, muscular strength, and kayaking performance compared to traditional kayak-specific HIIT regimen in well-trained kayak sprint athletes. We hypothesized that both HIIT protocols will improve the cardiorespiratory fitness adaptations of well-trained kayakers compared to a control group. Also, resistance-type HIIT will enhance both cardiorespiratory fitness and muscular strength simultaneously.

## Materials and Methods

### Participants

Twenty-four well-trained male kayakers (mean age=27 ± 4 years; height = 180 ± 2 cm; mass = 83 ± 5 kg; body fat = 9.4 ± 1.3%; years of experience = 11 ± 3 years) provided their written informed consent and volunteered to participate. All participants were members of the Iran national kayak sprint team and 18 were medalists of the Asian championships. Following screening for the presence of any unknown disease or conditions putting them at risk of adverse response to high-intensity exercise, participants were randomly assigned to paddling based HIIT (PHIIT), resistance-type HIIT (RHIIT), or a control group (CON). All procedures were in accordance with ethical principles of the Declaration of Helsinki and approved by the institutional review board of the University of Guilan and ethical committee of Sport Sciences Research Institute of Iran (approval ID: IR.SSRC.REC.1400.019).

### Overview of Experimental Protocol

In order to become oriented with all devices, testing procedures, and training protocols, all participants performed some familiarization visits to the laboratory at least 3 days before baseline measurement. Pre-testing of cardiorespiratory fitness and anaerobic power, along with cardiac structure and hemodynamic parameters as well as blood and biochemical parameters was conducted before the beginning of pre-season training, with post-testing held immediately after completion of the exercise programs. Prior to the beginning of training period, participants completed 3 sessions of a 30-min paddling time trial on a kayak ergometer (Dansprint, Hvidovre, Denmark) at a self-selected pace and an incremental paddling test to volitional fatigue. Participants also performed two upper body 30-s Wingate tests on a separate day to become familiar with these performance tests.

**FIGURE 1:**
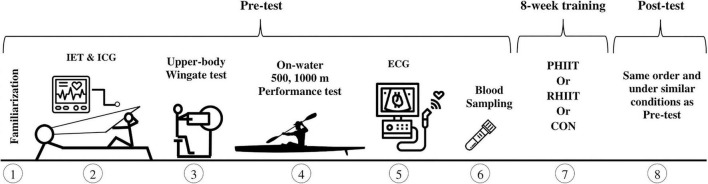
Overview of the experimental protocol. ECG, echocardiography; IET, incremental exercise test; ICG, impedance cardiography; PHIIT, paddling-based HIIT; RHIIT, resistance-type HIIT. Numbers in circles denote sequences of testing procedure and training period.

In the pre- and post-training, participants completed an incremental exercise test to determine peak oxygen uptake ($\dot{\text{V}}$O_2peak_) and related physiological variables. Time for which *v*$\dot{\text{V}}$O_2peak_ can be maintained (Tmax), upper-body Wingate test, one repetition maximum (1RM) in one-armed cable row, and 500 and 1,000-m on-water paddling performances were also evaluated on separate days. Cardiac structure and hemodynamics were evaluated before and after training. Also, body composition was determined using bioelectrical impedance analysis (Inbody 520, South Korea). All the aforementioned tests were completed in the morning, with 24 h of recovery separating each test. Figure 1 shows a schematic of the sequence of methods and order of the tests used in the present study.

### Incremental Exercise Test

Using a breath-by-breath gas collection system (Cosmed K4B2, Rome, Italy), participants performed an incremental paddling test on a kayak ergometer to determine $\dot{\text{V}}$O_2peak_, *v*$\dot{\text{V}}$O_2peak_, maximal ventilation ($\dot{v}$_E_@$\dot{\text{V}}$O_2peak_), respiratory frequency (*R*_f_@$\dot{\text{V}}$O_2peak_), and tidal volume ($\dot{V}$_T_@$\dot{\text{V}}$O_2peak_). The test began at 6 km⋅h^–1^ and workload increased 1 km⋅h^–1^ every 1 min until volitional exhaustion (Sheykhlouvand et al., 2016a,b, 2018a,b). The drag of the ergometer was adjusted according to the kayakers’ body mass as recommended by the producer to simulate on-water drag during paddling. When three or more of the following criteria were met, participants were considered to have reached their $\dot{\text{V}}$O_2peak_: (I) the $\dot{V}$O_2_ ceased to increase linearly despite the increase in workload and approached a plateau or decreased slightly with the last two values within ± 2 ml^–1^⋅kg^–1^⋅min^–1^; (II) a respiratory exchange ratio reached to 1.1 (Sheykhlouvand et al., 2016a,b, 2018a,b). The v$\dot{V}$O_2p*eak*_ (or Vmax) was defined as the minimal speed at which the athlete was paddling when $\dot{V}$O_2p*eak*_ occurred.

### Hemodynamic Function

The hemodynamic function was evaluated using a transthoracic electrical impedance cardiograph device (PhysioFlow, Manatec, France). This method has been validated and described by previous researches and is known as a reliable method at rest and exercise up to $\dot{\text{V}}$O_2max_ (Charloux et al., 2000; Richard et al., 2001). Two electrodes were placed on the neck, two at the xiphoid sternum, and one on each side of the chest as recommended by the manufacturer. Once a 20-s calibration was completed, hemodynamic values were recorded. At termination of incremental exercise, maximal values for HR (HR_max_), stroke volume (SV_max_), cardiac output ($\dot{Q}$_max_), end-diastolic volume (EDV), end-systolic volume (ESV), and ejection fraction (EF) were obtained.

### Upper-Body Wingate Test

Using a mechanically braked arm ergometer (891E; Monark, Vansbro, Sweden), participants completed a 30-s all-out effort to determine peak power output (PPO) and average power output. Participants were instructed to crank against the internal resistance of the ergometer as fast as possible and a load equivalent to 0.075 kg⋅kg^–1^ body mass (Forbes et al., 2014) was applied instantaneously (within 3 s). Participants were verbally encouraged to crank as fast as possible throughout the test. PPO and average power output were calculated using the devise software.

### Time for Which v$\dot{\text{V}}$O_2peak_ Can Be Maintained (Tmax)

After a warm-up comprising 5-min of paddling at 50% *v*$\dot{\text{V}}$O_2peak_, 5 min stretching, and another 5 min of paddling at 60% *v*$\dot{\text{V}}$O_2peak_, the paddling speed was increased to *v*$\dot{\text{V}}$O_2peak_ and participants exercised on kayak ergometer until exhaustion. The test was terminated volitionally by the subject if the desired speed could not be maintained and the time maintained at *v*$\dot{\text{V}}$O_2peak_ (Tmax) was recorded. Participants were verbally encouraged to paddle as long as possible. In the post-training, this test was repeated following identical procedures.

### One Repetition Maximum (1RM) in One-Armed Cable Row

1RM testing started with a warm-up consisting of 5 min of paddling on an ergometer (Dansprint, Hvidovre, Copenhagen, Denmark) at 6 km⋅h^–1^ followed by upper-body joint mobilization exercises. Following a 2-min recovery, participants performed one set of 6 repetitions with a load equal to 60% estimated 1RM followed by one set of 2–3 repetitions at 80% estimated 1RM (Seated pulley cable row machine, Technogym, Italy). Thereafter, to control the work rate accurately and simplify the matching equation that will be explained in section “Exercise Training Protocols,” the movement distance of the one-armed cable row was set at 1 meter (distance between the beginning and the end of motion) and participants performed 3–5 one-repetition sets only with one arm individually and with a 4-min recovery between sets to determine 1RM. The heaviest load that each subject could properly lift in a 1-m motion distance was considered to represent his 1RM (Ualí et al., 2012).

### Transthoracic Echocardiography

Using a Vivid E95 Ultra edition machine (GE Healthcare, Chicago, Illinois, United States) and according to the recent guidelines (Lang et al., 2015), transthoracic echocardiography was performed at rest and in a left lateral decubitus position. HR was continuously measured by a single-lead electrocardiogram. In the two-dimensional view, structural parameters were recorded with linear internal measurements of the left ventricle (LV) acquired in the parasternal long-axis view. Stroke volume, interventricular septal wall thickness (IVSWT), left ventricle mass (LVM), and left ventricle end-systolic and end-diastolic diameters (LVESd and LVEDd) were recorded. All echocardiographic studies were reviewed by the same cardiologist blinded to group allocation.

### Blood Sampling

Participants were sampled in the morning after an overnight fast exceeding 8 h. A 10-ml venous blood sample was collected in the pre- and post-training by venipuncture from an antecubital vein in the morning after an overnight fast exceeding 8 h. Seven milliliters of blood was immediately spun at 3,000 rpm for 15 min at 4°C and separated and stored at −80°C for measuring total testosterone and cortisol. Serum concentrations of total testosterone [Cayman Chemical, Ann Arbor, Michigan, United States; intra-assay coefficient of variation (CV) = 4.4%] and cortisol (Cayman Chemical, Ann Arbor, Michigan, United States; intra-assay CV = 6.7%) were determined by ELISA kits. Also, using an automated cell counter (Abacus C; Diatron, Budapest, Hungary), the remaining 3-ml blood sample was measured to record complete blood count. Also, plasma volume change was calculated using following equation (Rotstein et al., 1982):


$$\mathrm{Plasma}\mathrm{volume}\mathrm{change}=100\times \left[\frac{{\text{Hb}}_{\text{pre}}}{{\text{Hb}}_{\text{post}}}\times \frac{\left(1-{\text{Hct}}_{\text{post}}\times 10^{-2}\right)}{\left(1-{\text{Hct}}_{\text{pre}}\times 10^{-2}\right)}\right]-100$$

Hb_pre_ = hemoglobin concentration before the exercise,

Hb_post_ = hemoglobin concentration after the exercise,

Hct_pre_ = hematocrit value before the exercise (%),

Hct_post_ = hematocrit value after the exercise (%).

### On-Water Exercise Performance

In the pre- and post-training, participants completed 500- and 1,000-m on-water paddling tests over three consecutive days. The day first was dedicated to the 500-m test; participants then rest during the second day, and they completed the 1,000-m test on the third day. Prior to testing, they completed a standardized warm-up according to Borges et al. (2015). Each participant performed two trials of 500-m test and two trials of 1,000-m test interspersed with 1 h of passive recovery. Time was recorded using two synchronized stopwatches (Interval 2,000 XC Track and Field watch, Nielsen-Kellerman, Delaware, Pennsylvania, United States) and the best times were used for analysis. The tests were performed on flat water with an average tail wind of ∼3.2 m⋅s^–1^ at an ambient temperature of ∼23°C. The condition was almost the same during pre- and post-training.

### Exercise Training Protocols

Approximately 48 h after the last baseline measurement, participants underwent 8-weeks of kayak ergometer training, on-water paddling program, or one-armed cable row. Participants undergoing HIIT (PHIIT and RHIIT) performed three HIIT sessions and three traditional on-water paddling sessions each week. In PHIIT, the subjects performed six intervals at 100% *v*$\dot{\text{V}}$O_2peak_ with training volume varying each week (60, 70, 75, 75, 75, 75, 70, and 60)%Tmax from first to eighth week, respectively, using a 1:1 work to recovery ratio. Traditional on-water paddling sessions consisted of 60 min of paddling at 70–80% HRmax (55–75% $\dot{\text{V}}$O_2max_; Haff and Triplett, 2016).

The participants performing RHIIT completed one-armed cable row training matched with PHIIT with respect to total work and training duration. As the training mode was one-armed, the hands were alternated during efforts. For matching the total work, work rate at 100% *v*$\dot{\text{V}}$O_2peak_ [Watts (W)] was recorded from the kayak ergometer. Each W is equal to 1 Joule⋅s^–1^ where each Joule is a result of force [Newton (N)] multiplied by distance [meter (M)] leaving:


$$\text{Power }\left(\text{W}\right)=\frac{\text{Force }\left(\text{N}\right)\times \mathrm{Distance}\left(\text{M}\right)}{\text{Time }\left(\text{sec}\right)}$$

Considering time commitment of %Tmax (sec) leads to the following equation:


Work(Force [N]×Distance [M])=Power (W)×Time (sec [%Tmax])

By multiplying work (Newton-force Meter) by 0.10197, we can convert it to Kilogram-force Meter and the equation would be:


Work (Force[kg]×Distance [M])=Power (W)×Time (sec [%Tmax])×0.10197

Subsequently, 1RM of one-armed cable row in a 1-m motion distance was evaluated and target 1RM [%1RM (kg)] was identified. The PHIIT regimen requires a high-volume training with respect to the time (%Tmax), the RHIIT and PHIIT were performed in the strength endurance phase of the kayakers’ yearly training program where the intensity of repetitions is low to moderate (50–75% 1RM) and the volume is high (Haff and Triplett, 2016). Then, the force (kg) that must be carried in 1-m distance divided by target 1RM [50% 1RM (kg)] and the number of repetitions in one-armed cable row in 1-m distance was specified. On this occasion, as the distance of motion in one-armed cable row is 1 m, the value of force (kg) and work [force (kg) multiplied by distance (1)] would be the same and the number of repetitions will be as follow:


$$\mathrm{Number}\mathrm{of}\mathrm{repetitions}=\frac{\text{Force }\left(\text{kg}\right)}{50\%1\text{RM }\left(\text{kg}\right)}$$

Considering that each athlete had his own 1RM and Tmax, the number of repetitions was specialized and varied among the participants.

With such a matching method, the total work performed during PHIIT and RHIIT would be the same. The key point is the difference between imposed force by each stroke (during paddling HIIT) and each repetition (during resistance HIIT) and the total number of strokes or reps during the working time (Tmax). The difference is that the number of strokes during paddling HIIT is more than the number of repetitions during RHIIT but the imposed force in each repetition in RHIIT is greater than that of each paddling stroke in PHIIT.

The participants in CON performed six sessions of on-water kayak paddling per week including 60 min of traditional endurance paddling at 70–80% HR_max_. Also, all three groups performed 1 d/wk of Fartlek training [45 min of long slow distance run (LSD)] and 2 sessions per week of weight training consisting of 3 sets of 8–12 repetitions at 70% 1RM including bench pull, bench press, seated row, bicep curl, military press, pulley pushdowns and trunk rotation) and push-ups, sit-ups, and pull-ups.

### Dietary Control

To avoid potential confounding of the results mediated by taking supplements and stimulants, participants were directed to continue the same habitual nutrition intake during the experiment. Consuming the same diet 48 h prior to and post-training assessment was encouraged. In addition, subjects were asked to refrain from participating in vigorous activity and to avoid the consumption of caffeinated food and beverages in the 24-h period prior to testing.

### Statistical Analyses

Sample size for three groups (*N* = 8) was calculated using G*Power software (Faul et al., 2007) and Statistical analyses were performed using SPSS, version 25.0 (Statistical Package for Social Science, Chicago, IL). Results were expressed as mean ± *SD*. The Shapiro-Wilk test was used to test the normality and Levene’s test was used to assess homogeneity of variances. The data were analyzed using a two-factor mixed analysis of variance (ANOVA) with the between factor “group” (PHIIT, RHIIT, and CON) and repeated factor “time” (pre-training, post-training). Significant interactions or main effects were subsequently analyzed using a Tukey’s honestly significant difference *post-hoc* test. One-way ANOVA was used to analyze difference between changes in plasma volume in different groups. Pearson product–moment correlations were used to examine relationships between variables. Effect size was calculated using Cohen’s *d* (d). Alpha level was set at 0.05.

## Results

### Change in Maximal Gas Exchange Variables, Power Output and One Repetition Maximum

**FIGURE 2:**
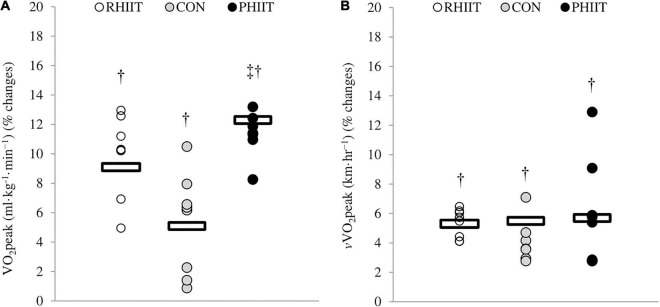
Effects of paddling-based HIIT (PHIIT), resistance-type HIIT (RHIIT), and on-water kayak sprint (CON) training on changes in **(A)** $\dot{\text{V}}$O_2peak_, and **(B)** *v*$\dot{\text{V}}$O_2peak_. Circles show individual percentage change from baseline and horizontal bars show mean group percentage change from baseline (X-axes). *N* = 8 for each group. ^†^Significantly different vs. pre-training (*p* ≤ 0.05). ^‡^Significantly different vs. control group (*p* ≤ 0.05).

No pre-training difference was observed between groups for these physiological parameters. After the 8-week training program, a significant time-regimen interaction (*p* = 0.04) was found in $\dot{\text{V}}$O_2peak_. As shown in Figure 2A, the change in $\dot{\text{V}}$O_2peak_ (ml⋅kg^–1^⋅min^–1^) in response to PHIIT was significantly greater compared to CON (*p* = 0.03, *d* = 2.02). $\dot{\text{V}}$O_2peak_ was significantly increased in PHIIT (Post: 54.35 ± 3.65 vs. Pre: 48.39 ± 3.91 ml⋅kg^–1^⋅min^–1^,%Δ = 12.3, *p* = 0.0006, *d* = 1.6), RHIIT (Post: 52.31 ± 4.97 vs. Pre: 47.59 ± 4.48 ml⋅kg^–1^⋅min^–1^, %Δ = 9.1, *p* = 0.002, *d* = 1.0), and CON (Post: 48.31 ± 2.13 vs. Pre: 45.95 ± 2.73 ml⋅kg^–1^⋅min^–1^, %Δ = 5.1, *p* = 0.003, *d* = 0.9) compared with pre-training.

After the 8-week training period, *v*$\dot{\text{V}}$O_2peak_ significantly increased in PHIIT (Post: 18.2 ± 0.5 vs. Pre: 17.2 ± 0.9 km⋅h^–1^, %Δ = 5.7, *p* = 0.001, *d* = 1.2), RHIIT (Post: 17.8 ± 0.5 vs. Pre: 16.9 ± 0.6 km⋅h^–1^, %Δ = 5.3, *p* = 0.0004, *d* = 1.5), and CON (Post: 17.7 ± 0.4 vs. Pre: 16.8 ± 0.4 km⋅h^–1^, %Δ = 5.5, *p* = 0.006, *d* = 2.5) compared with pre-training, but there was no time-regimen interaction (*p* = 0.91) (Figure 2B).

Maximal $\dot{V}$_E_ significantly increased from pre- to post-training in PHIIT and RHIIT (*p* = 0.003, *d* = 1.4 and 1.2, respectively) but not in CON and there was no between-group difference for this variable (*p* = 0.65). In addition, a significant increase occurred in *R*_f_@$\dot{\text{V}}$O_2peak_ from pre- to post-training in PHIIT and RHIIT (*p* = 0.001 and 0.004, *d* = 2.0 and 0.8, respectively) but not CON. Also, the change in *R*_f_@$\dot{\text{V}}$O_2peak_ in response to RHIIT was significantly greater compared to the change in CON (*p* = 0.03, *d* = 1.2). There was no change in $\dot{V}$_T_@$\dot{\text{V}}$O_2peak_ across time (*p* > 0.05).

There were no between-group differences for PPO or average PO (*p* = 0.64 and 0.25, respectively). PPO significantly increased after training in PHIIT, RHIIT, and CON (*p* = 0.0003, 0.003, and 0.002, *d* = 1.1, 1.0, and 0.3, respectively). Average PO was significantly increased in response to PHIIT (*p* = 0.001, *d* = 0.7) and RHIIT (*p* = 0.05, *d* = 0.5) compared with pre-training but not CON (Table 1).

**TABLE 1:** Change in gas exchange indices, power output and 1RM in response to training.

		Group	
	PHIIT	RHIIT	CON
**$\dot{V}$_E_@$\dot{\text{V}}$O_2peak_ (l.min^–1^)**			
Pre Post %Δ	131.81 (10.80) 151.82 (15.61) † + 15.2	130.85 (8.09) 143.88 (13.63) † + 9.9	137.73 (12.19) 144.82 (10.81) + 5.1
**$\dot{V}$_T_@$\dot{\text{V}}$O_2peak_ (l.b^–1^)**			
Pre Post %Δ	2.35 (0.17) 2.34 (0.23) –0.04	2.24 (0.35) 2.27 (0.33) + 1.3	2.35 (0.29) 2.36 (0.21) + 0.04
***R*_f_@$\dot{\text{V}}$O_2peak_ (b.min^–1^)**			
Pre Post %Δ	56.17 (2.66) 62.25 (3.35) † + 10.8	59.06 (6.57) 63.58 (4.02) † + 7.6 ‡	55.36 (4.78) 57.75 (5.54) + 4.3
**PPO (W)**			
Pre Post %Δ	530.95 (35.0) 570.10 (42.4) † + 7.3	512.16 (49.3) 564.83 (52.8) † + 10.3	514.70 (72.1) 535.67 (67.1) + 4.1
**Average PO (W)**			
Pre Post %Δ	396.71 (51.8) 430.07 (56.4) † + 8.4	387.57 (50.8) 409.95 (46.2) † + 10.5	374.20 (37.14) 379.50 (27.97) + 1.4
**1RM in OACR with right hand (kg)**			
Pre Post %Δ	69.1 (5.7) 71.9 (3.9) + 4.0	67.1 (2.9) 72.2 (2.8) † + 7.6	68.9 (4.6) 72.0 (4.1) + 4.5
**1RM in OACR with left hand (kg)**			
Pre Post %Δ	66.6 (5.2) 69.6 (4.2) + 4.5	64.5 (2.8) 69.4 (3.0) † + 7.5	66.1 (3.6) 68.7 (3.4) + 3.9

*Values are means (± SD).*

*PPO, peak power output; $\dot{V}$_E_, ventilation; $\dot{V}$_T_, tidal volume; R_f_, respiratory frequency; 1RM, one repetition maximum; OACR, one-armed cable row; N, 8 for each group.*

*^†^Significantly greater than pre-training value (p < 0.05). ^‡^Significantly different change compared with CON group (p < 0.05).*

*N = 8 for each group.*

Maximum strength expressed as 1RM in one-armed cable row significantly improved over time for right and left hand in response to RHIIT (*p* = 00008, 0.00001; *d* = 1.7, 1.6, respectively). No training-induced increase in this variable was observed in PHIIT and CON with no between-group difference for the magnitude of changes pre- to post-training.

### Change in Biochemical Outcomes in Response to Training

**FIGURE 3:**
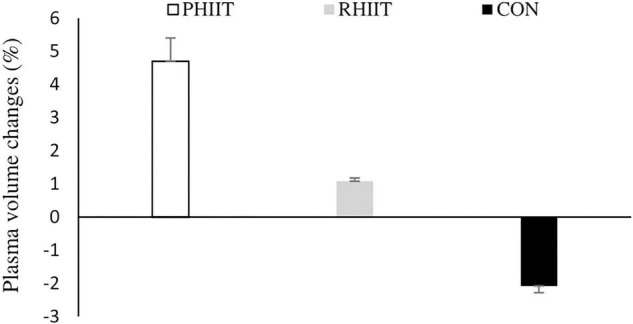
Effects of paddling-based HIIT (PHIIT), resistance-type HIIT (RHIIT), and on-water kayak sprint (CON) training on plasma volume changes. *N* = 8 for each group.

There was no pre-training difference (*p* > 0.05) observed between groups for any biochemical variables. Table 2 presents the resting hormone concentrations and hematological changes in response to the 8-week training period. Compared to pre-training, a significant increase was observed in total testosterone concentration in PHIIT and RHIIT (*p* = 0.02 and 0.03, *d* = 1.0 and 0.7, respectively). No significant differences were observed among groups (*p* = 0.35). There was a training-induced increase in Testosterone/Cortisol ratio (T/C ratio) in PHIIT and RHIIT (*p* = 0.05, *d* = 0.8) but not in CON (*p* > 0.05). Also, there was no between-group difference for T/C ratio (*p* = 0.76). Results showed no change in cortisol, hemoglobin (Hb), red blood cell (RBC), or hematocrit (Hct) in response to training (*p* > 0.05). Plasma volume changes over time were 4.7, 1.1, and –2.1% in PHIIT, RHIIT, and CON groups, respectively (Figure 3). No between-group difference was found for change in plasma volume (*p* = 0.13).

**TABLE 2:** Change in biochemical outcomes in response to training.

		Group	
	PHIIT	RHIIT	CON
**TT (μg⋅dl^–1^)**			
Pre Post %Δ	0.588 (0.14) 0.716 (0.10) † + 21.7	0.624 (0.14) 0.733 (0.15) † + 17.4	0.586 (0.09) 0.599 (0.11) + 2.2
**Cortisol (μg⋅dl^–1^)**			
Pre Post %Δ	20.12 (1.88) 19.47 (2.28) -3.3	19.97 (5.10) 18.55 (4.08) -7.6	18.65 (4.90) 18.43 (4.67) -1.1
**T/C ratio**			
Pre Post %Δ	0.029 (0.00) 0.037 (0.00) † + 27.6	0.032 (0.00) 0.042 (0.01) † + 31.2	0.034 (0.01) 0.035 (0.01) + 2.9
**RBC (Mill⋅mm^–3^)**			
Pre Post %Δ	5.59 (0.24) 5.42 (0.37) -3.1	5.48 (0.29) 5.60 (0.49) + 2.1	5.72 (0.44) 5.73 (0.30) + 0.1
**Hb (g⋅dl^–1^)**			
Pre Post %Δ	15.70 (0.72) 15.38 (1.09) -2.0	15.23 (1.43) 15.23 (1.38) 0.0	15.36 (1.44) 15.65 (1.35) + 1.8
**Hct (%)**			
Pre Post %Δ	47.13 (1.51) 46.26 (2.20) -1.8	46.78 (2.02) 46.48 (2.29) -0.6	47.11 (2.29) 47.01 (2.56) -0.2

*Values are means (± SD).*

*Hb, hemoglobin; Hct, hematocrit; RBC, red blood cell; TT, total testosterone; T/C, testosterone/cortisol. N, 8 for each group.*

*^†^Significantly greater than pre-training value (p < 0.05).*

*N, 8 for each group.*

### Change in Cardiac Structure and Hemodynamics in Response to Training

No pre-training difference was observed between groups for cardiac morphology or hemodynamics. As indicated in Table 3, SV_max_ significantly increased after training in response to PHIIT (*p* = 0.006, *d* = 1.0) and RHIIT (*p* = 0.003, *d* = 1.1) but there was no change in CON (*p* = 0.23). $\dot{Q}$_max_ showed a significant main effect for time in PHIIT (*p* = 0.006, *d* = 1.2), RHIIT (*p* = 0.001, *d* = 1.7), and CON (*p* = 0.05, *d* = 0.5), but there was no time-regimen interaction for $\dot{Q}$_max_ (*p* = 0.41). EDV and EF significantly increased from pre- to post-training in PHIIT (*p* = 0.004 and 0.05, *d* = 1.2 and 0.9, respectively) and RHIIT (*p* = 0.002 and 0.03, *d* = 1.3 and 0.5, respectively) groups, but not in CON. Data showed no change in ESV or HR_max_ across time in groups.

**TABLE 3:** Change in cardiac structure and hemodynamics in response to training.

		Group	
	PHIIT	RHIIT	CON
**SV_max_ (ml⋅b^–1^)**			
Pre Post %Δ	131.62 (17.2) 147.25 (10.5) † + 11.9	128.12 (15.2) 143.62 (12.3) † + 12.1	127.37 (17.5) 132.62 (13.4) + 4.1
**HR_max_ (b⋅min^–1^)**			
Pre Post %Δ	185.6 (4.4) 187.3 (3.6) + 0.9	191.2 (10.8) 193.6 (5.4) + 1.2	187.0 (4.5) 191.1 (4.8) + 2.2
**$\dot{Q}$_max_ (l⋅min^–1^)**			
Pre Post %Δ	24.5 (3.2) 27.5 (1.7) † + 12.2	24.3 (2.0) 27.7 (1.9) † + 13.9	23.8 (3.5) 25.4 (2.9) † + 6.7
**EDV (ml)**			
Pre Post %Δ	171.4 (14.8) 186.7 (10.3) † + 8.9	170.7 (14.6) 187.2 (9.9) † + 9.6	169.6 (4.7) 174.8 (3.4) + 3.0
**ESV (ml)**			
Pre Post %Δ	40.8 (2.5) 39.7 (2.1) -2.8	42.6 (1.43) 42.3 (1.38) -0.7	42.2 (4.1) 42.1 (4.2) -0.2
**EF (%)**			
Pre Post %Δ	75.7 (3.6) 79.8 (1.5) † + 5.4	73.8 (3.5) 77.6 (3.2) † + 5.1	74.7 (2.29) 75.6 (2.56) + 1.2
**SV_*rest*_ (ml⋅b^–1^)**			
Pre Post %Δ	80.3 (5.0) 84.9 (6.7) † + 5.7	78.2 (4.4) 82.3 (4.0) † + 5.2	81.2 (5.8) 84.3 (4.6) † + 4.8
**IVSWT (mm)**			
Pre Post %Δ	8.03 (0.6) 8.05 (0.6) + 0.2	8.07 (0.8) 8.09 (0.7) + 0.2	7.93 (0.4) 7.93 (0.5) + 0.0
**LVM (g)**			
Pre Post %Δ	180.4 (26.0) 180.7 (25.4) + 0.1	181.6 (29.1) 182.0 (28.6) + 0.2	184.3 (36.0) 184.5 (37.1) + 0.1
**LVESd (mm)**			
Pre Post %Δ	42.0 (4.0) 40.1 (3.2) † -4.7	41.7 (2.9) 39.7 (2.7) † -5.0	41.0 (3.0) 39.2 (3.4) † -4.6
**LVEDd (mm)**			
Pre Post %Δ	53.1 (3.8) 54.3 (3.6) + 2.2	52.1 (4.3) 52.7 (4.1) + 1.1	53.7 (3.1) 54.3 (3.9) + 1.1

*Values are means (± SD).*

*EF, ejection fraction; EDV, end-diastolic volume; ESV, end-systolic volume; HR, heart rate; IVSWT, interventricular septal wall thickness; LVM, left ventricular mass; LVESd, left ventricular end-systolic diameters; LVEDd, left ventricular end-diastolic diameters; $\dot{Q}$, cardiac output; SV, stroke volume. N, 8 for each group.*

*^†^Significantly greater than pre-training value (p < 0.05).*

*N, 8 for each group.*

Resting values of SV and LVESd were significantly increased across time in PHIIT (*p* = 0.01 and 0.01, *d* = 0.7 and 0.5, respectively), RHIIT (*p* = 0.01 and 0.01, *d* = 0.9 and 0.7, respectively), and CON (*p* = 0.005 and 0.01, *d* = 0.6 and 0.5, respectively). No significant difference was observed for resting values of IVSWT, LVM, and left LVEDd compared with pre-training (*p* > 0.05). No between group difference was observed in the change in cardiac dimensions over time (Table 3).

### Change in on-Water Kayak Sprint Performance in Response to Training

**FIGURE 4:**
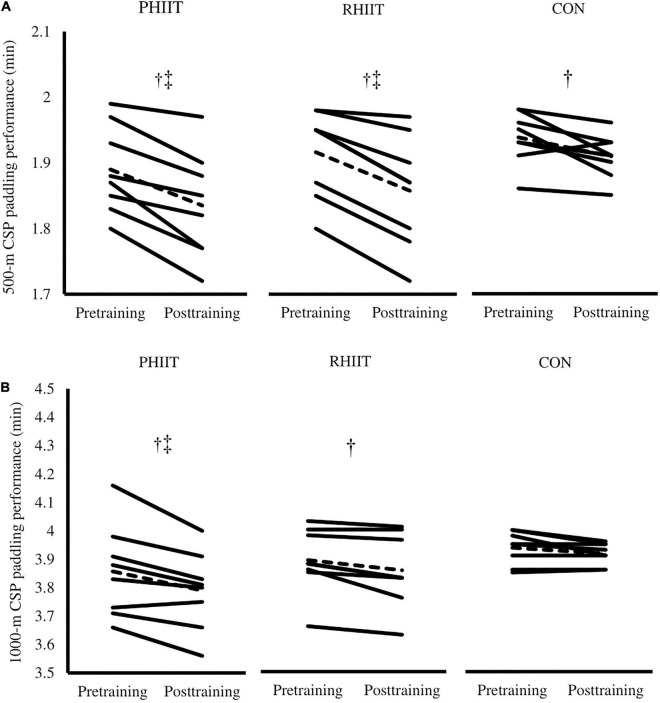
Effects of paddling-based HIIT (PHIIT), resistance-type HIIT (RHIIT), and on-water kayak sprint (CON) training on **(A)** 500-m on-water kayak sprint paddling performance, and **(B)** 1,000-m on-water kayak sprint paddling performance. N = 8 for each group. Each line represents an individual participant and the dotted line represents the mean response. ^†^Significantly different compared with pre-training (*p* ≤ 0.05). ^‡^Significantly different vs. control group (*p* ≤ 0.05).

No pre-training difference (*p* > 0.05) was observed between groups for 500- and 1,000-m paddling performance. Figures 4A,B show changes in 500- and 1,000-m kayak sprint performance from pre- to post-training. A significant time-regimen interaction (*p* = 0.05) was found in 500-m paddling performance as the change in 500-m paddling performance in response to PHIIT and RHIIT was significantly greater compared to the change in CON (*p* = 0.02, *d* = 0.6; and *p* = 0.05, *d* = 0.4, respectively). In response to training, 500-m paddling time significantly decreased in PHIIT (Post: 109.1 ± 5.1 vs. Pre: 112.5 ± 4.1 s, Δ≈ –3.4 s, *p* = 0.0008, *d* = 0.7), RHIIT (Post: 110.3 ± 4.9 vs. Pre: 114.0 ± 3.9 s, Δ≈ –3.7 s, *p* = 0.0006, *d* = 0.8), and CON (Post: 114.7 ± 2.1 vs. Pre: 116.6 ± 2.3 s, Δ≈ –1.9 s, *p* = 0.02, *d* = 0.8).

As shown in Figure 4B, a significant time-regimen interaction (*p* = 0.04) was found in 1,000-m paddling performance. The change in 1,000-m paddling performance in response to PHIIT was significantly greater compared to the change in CON (*p* = 0.05, *d* = 0.9). The 1,000-m paddling time was significantly decreased in PHIIT (Post: 227.1 ± 8.6 vs. Pre: 231.0 ± 7.4 s, Δ≈ –3.9 s, *p* = 0.0001, *d* = 0.5) and RHIIT (Post: 231.6 ± 7.8 vs. Pre: 233.7 ± 6.9 s, Δ≈ –2.1 s, *p* = 0.0004, *d* = 0.6) but not in CON (Post: 235.1 ± 2.2 vs. Pre: 236.0 ± 3.9 s, Δ≈ –0.9 s, *p* = 0.17) compared with pre-training.

Also, 500- and 1,000-m paddling performances were negatively correlated to $\dot{\text{V}}$O_2peak_ in pre- and post-training (*r* = –0.92, *p* = 0.00001; *r* = –0.91, *p* = 0.000001, respectively) and post-training (*r* = –0.91, *p* = 0.00001; *r* = –0.81, *p* = 0.000001, respectively).

## Discussion

This study examined changes in cardiorespiratory fitness, hemodynamics, exercise performance, and muscular strength in response to resistance training HIIT matched with the lowest velocity that elicits $\dot{\text{V}}$O_2peak_ (100% *v*$\dot{\text{V}}$O_2peak_) and compared the adaptations vs. paddling-based interval exercise at 100% *v*$\dot{\text{V}}$O_2peak_ and traditional endurance paddling in well-trained kayak sprint athletes. The major findings from this study were that 8 weeks of either RHIIT or PHIIT improved cardiorespiratory fitness and kayak sprint performance, resting values of cardiac dimensions, maximum stroke volume and cardiac output, to a similar extent. In the case of $\dot{\text{V}}$O_2peak_ and 500- and 1,000-m paddling performances, these responses were superior to traditional continuous paddling. Also, RHIIT enhanced maximal strength and cardiorespiratory fitness adaptations simultaneously.

As a primary determinant of aerobic endurance, $\dot{\text{V}}$O_2peak_ has been identified as the primary contributor in improving on-water kayak sprint paddling performance after HIIT (Dolci et al., 2020; Gharaat et al., 2020). This contention is verified by our data showing enhanced paddling performances that was consequent with an increase in $\dot{\text{V}}$O_2peak_ as 500- and 1,000-m paddling performances were negatively correlated to $\dot{\text{V}}$O_2peak_ in pre- and post-training. All training groups revealed an increase in $\dot{\text{V}}$O_2peak_ compared to pre-training, although this increase was superior in response to PHIIT vs. control. It is generally accepted that improvements in $\dot{\text{V}}$O_2peak_ may occur through increases in both oxygen delivery and/or its utilization within the active organs (Sheykhlouvand et al., 2018a,b). Although RBC and Hb remained unchanged pre- to post-training, $\dot{Q}$_max_ significantly enhanced in all training groups indicating improved oxygen crying capacity. The major purpose of the increase in cardiac output is to meet the muscles’ increased demand for oxygen. In fact, it is likely that $\dot{\text{V}}$O_2max_ is ultimately limited by the inability of cardiac output to increase further (Kenney et al., 2012). Our findings support the study of Astorino et al. (2017) and Mahjoub et al. (2019) who reported increased cardiac output in response to HIIT in active men and women. In these studies, an increase in maximal SV_max_ led to the increase in $\dot{Q}$_max_ which supports our results. Improved SV_max_ in response to HIIT can be attributed to increased EDV, increased force of contraction, and/or an increase in blood volume (Warburton et al., 2004). Nevertheless, neither PHIIT nor RHIIT modified ESV or cardiac morphology, leading to the conclusion that in elite athletes, increased contractile force through structural changes of the heart is not able to improve EDV following PHIIT and RHIIT. However, resting values of LVESd significantly decreased after the training period (Table 3) and SV_*rest*_ significantly increased in all training groups showing improved ventricular contractility. Significantly increased EDV and SV_max_ in PHIIT could in part be an explanation of the superior $\dot{\text{V}}$O_2peak_ observed compared to CON. One of the likely mechanisms explaining enhanced EDV is an increase in plasma volume via interval training which occurred even following short-term HIIT (3–6 sessions) (Warburton et al., 2004) causing an increase in blood volume and thus greater diastolic filling (Astorino et al., 2017). However, our results showed no between-group difference for plasma volume changes pre- to post-training. Despite an increase in both SV_max_ and EDV, we showed an increase in EF indicating greater changes in SV_max_ compared to EDV. This could be influenced by factors other than increased blood volume and diastolic filling. In support of this, Kenney et al. (2012) mentioned that contractility can increase by increasing sympathetic nerve stimulation or circulating catecholamines (epinephrine, norepinephrine), or both. Also, excitation-contraction coupling in cardiomyocytes which is susceptible to change by exercise training can be enhanced through the faster systolic rise and faster diastolic decay of the Ca^2+^ transient, with the magnitude of contractility corresponding to the extent of cell shortening and relaxation rates (Wisløff et al., 2009). An improved force of contraction can increase SV with or without an increased EDV by increasing the ejection fraction.

During exercise, the cardiovascular and respiratory systems operate as an integrated “machine” for the transport of respired gases (McConnell, 2013). Our data show an increase in $\dot{V}$_E_@$\dot{\text{V}}$O_2peak_ following both HIIT interventions. This increase can be attributed to enhanced *R*_f_@$\dot{\text{V}}$O_2peak_ as $\dot{V}$_T_@$\dot{\text{V}}$O_2peak_ remained unchanged pre- to post-HIIT. At lower intensities, an increase in both *R*_f_ and $\dot{V}$_T_ is responsible for the enhanced $\dot{V}$_E_. However, at higher intensities, the respiratory muscles become actively involved, and respiratory muscle fatigue may develop (Sheykhlouvand et al., 2018a) leading to rapid shallow breathing, a plateau in $\dot{V}$_T_, and consequent steep rise in *R*_f_ to meet the need for an escalating $\dot{V}$_E_ (McConnell, 2013).

In accordance with our hypothesis, maximal strength increased in response to RHIIT when expressed as 1RM in one-armed cable row in both right and left hands. Neurological adaptations in the early stages of resistance training, along with enhanced muscle hypertrophy by continuing the training over weeks, are the main contributing factors in strength gain following resistance training as classically proposed (Deschenes, 1989; Chesley et al., 1992).

PPO and average PO were significantly increased in response to both HIIT modalities compared with pre-training, but not in CON. However, the magnitude of these improvements was not different between PHIIT and RHIIT indicating beneficial effects of both protocols. These findings support other investigations reporting increases in peak and mean anaerobic power in response to different HIIT regimens (Farzad et al., 2011; Sheykhlouvand et al., 2016b; Dolci et al., 2020; Hoffmann et al., 2020). Dolci et al. (2020) stated that only 2 weeks of HIIT in active men increases the discharge rate of high-threshold motor units and improves power output. Sheykhlouvand et al. (2018b) reported that an increased muscle buffering capacity may in part be responsible for the enhanced peak and mean power output in response to 4 weeks of training in elite athletes. Increased total creatine content of muscle and a significant increase in type II fiber size are other possible explanations for these changes (Hoffmann et al., 2020).

Both PHIIT and RHIIT protocols increased total testosterone levels and T/C ratio, but there was no change in serum cortisol. To determine the physiological strain of the training, the T/C ratio is frequently used as an indicator of catabolic-anabolic balance (Farzad et al., 2011; Sheykhlouvand et al., 2016a). Hence, the observed improvements following both HIIT protocols may indicate anabolic adaptations. These results support our previous findings in professional canoe polo paddlers in which paddling-based HIIT with incremental volume and intensity (60 s paddling at 100–130% *v*$\dot{\text{V}}$O_2peak_; 1:3 work to recovery) improves T/C ratio. In addition, Farzad et al. (2011) demonstrated increases in T/C ratio following HIIT (6 × 35-m sprint running with 10 s rest between reps). The increased T/C ratio may be attributed to the enhanced serum levels of TT as cortisol remained unchanged over time. Potential adaptations in TT synthesis and the secretory capacity of the Leydig cells could be possible explanations for our findings (Kraemer and Ratamess, 2005).

A limitation of this study was an inability to strictly monitor dietary practices of athletes during training. Moreover, we only recruited men, and our results cannot be applied to women competing in kayak sprinting. Our results only apply to our specific HIIT regimens, and it is unknown if similar adaptations would occur in response to higher volume HIIT or low volume sprint interval training. Although the environmental conditions were mostly similar during pre- and post-training, there was a slight difference in tail wind and ambient temperature pre- to post-training. We did not evaluate the water temperature, but the values were mostly identical within the period when we performed the experiment.

In conclusion, the results of this study showed that PHIIT and RHIIT similarly improve $\dot{\text{V}}$O_2peak_, maximal values of cardiac output and stroke volume, and resting values of cardiac dimensions in kayak sprint athletes. Results indicated that the improved 500 and 1,000-m on-water kayak sprint paddling performance following PHIIT and RHIIT are associated with the enhanced cardiorespiratory adaptations. Moreover, an elevated T/C ratio suggests that both HIIT protocols induce an anabolic-type hormonal adaptation indicating positive responses to training. Similar cardiorespiratory fitness increases following PHIIT and RHIIT could be justified by the similarity in total work performed during both protocols. Considering that the adaptations in response to RHIIT and PHIIT were mostly identical, kayak sprint athletes and their coaches can use either type of program to elicit improvements in exercise performance, cardiorespiratory fitness, and anabolic profile. Given that RHIIT effectively improved both cardiorespiratory fitness and maximal strength, this method could serve as a novel time-efficient strategy to simultaneously improve both qualities in well-trained kayak sprint athletes.

## Data Availability Statement

The raw data supporting the conclusions of this article will be made available by the authors, without undue reservation.

## Ethics Statement

The studies involving human participants were reviewed and approved by Sport Sciences Research Institute of Iran (approval ID: IR.SSRC.REC.1400.019). The patients/participants provided their written informed consent to participate in this study.

## Author Contributions

MS contributed to project administration, conceptualization, methodology, visualization, and investigation. HA supervised the project, methodology, and data analysis and contributed to conceptualization, reviewing, and editing of the manuscript. TA and KS contributed to reviewing, and editing of the manuscript. All authors contributed to the article and approved the submitted version.

## Conflict of Interest

The authors declare that the research was conducted in the absence of any commercial or financial relationships that could be construed as a potential conflict of interest.

## Publisher’s Note

All claims expressed in this article are solely those of the authors and do not necessarily represent those of their affiliated organizations, or those of the publisher, the editors and the reviewers. Any product that may be evaluated in this article, or claim that may be made by its manufacturer, is not guaranteed or endorsed by the publisher.

## Figures and Tables

**Table:** 

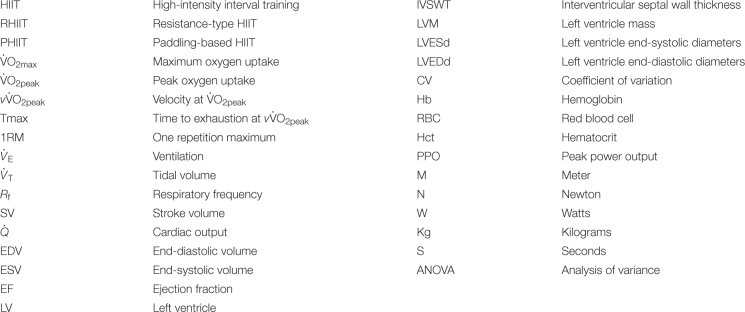

## Glossary

1
www.canoeicf.com
